# Do partnered male aphrodisiac users engage in higher sexual risk behaviors related to sexually transmitted infections than single men? Evidence from a survey conducted in Kinshasa, Democratic Republic of the Congo

**DOI:** 10.3389/fpubh.2026.1898629

**Published:** 2026-08-14

**Authors:** Didier Mbombo Ndombe, José Mobhe Agbada Mangalu, Manuel Ferdinand Manun'Ebo

**Affiliations:** 1 Centre for Demographic Research, Université catholique de Louvain, Louvain-la-Neuve, Belgium; 2 Centre Interdisciplinaire pour le Développement et l'Education Permanente, Kinshasa, Democratic Republic of Congo; 3 Ecole des Sciences de la Population et de Développement, Université de Kinshasa, Kinshasa, Democratic Republic of Congo; 4 Institut Superieur des Techniques Medicales de Lubumbashi, Lubumbashi, Democratic Republic of Congo

**Keywords:** aphrodisiac, human immunodeficiency virus, Kinshasa, partnered men, risky sexual behavior, sexual intercourse, sexually transmitted infections

## Abstract

Drugs meant to help with erectile dysfunction are more often used for fun and to enhance sexual performance. However, the non-medical use of these medications may encourage risky sexual behavior and raise the chances of getting sexually transmitted infections, such as Human Immunodeficiency Virus. In this study, we explore the association between the aphrodisiac use and the risky sexual behavior among men aged 15–49 years in Kinshasa, Democratic Republic of the Congo. This study is a secondary analysis of data from 654 men aged 15–49 years in Kinshasa, regardless of sexual orientation. The dataset includes detailed information on socio-demographic characteristics, the use of aphrodisiac substances, and potential exposure to sexually transmitted infections. To analyze the relationship between the use of aphrodisiacs and risky sexual behavior, linear probability models were utilized. Furthermore, to assess their association with risky sexual behaviors, the study investigated the interactions between aphrodisiac use and a few factors, including marital status, and age. The main findings state that among the 654 respondents selected, 48.7% reported using aphrodisiacs in the past 12 months [95% CI: 44.95–52.61]. A linear probability model revealed that the consumption of aphrodisiacs is associated with an increased risk of engaging in risky sexual behavior [aCoef = 0.22 (95% CI: 0.17–0.28, *p* < 0.001)]]. Moreover, the combined effect of aphrodisiac use and being in a union [aCoef = 0.34 (95% CI: 0.19–0.49)]] or being at least 35 years old [aCoef = 0.28 (95% CI: 0.13–0.43)] further increases this risk. In summary, the study shows a higher risky sexual behavior among men using aphrodisiacs in Kinshasa—particularly among those currently in a cohabiting union—and underscores the urgency of implementing targeted prevention strategies.

## Introduction

In 2021, an estimated 38,4 million people were living with Human Immunodeficiency Virus (HIV) worldwide, with new infections totaling 1.5 million that year. Tragically, 650 000 individuals lost their lives due to HIV/AIDS-related illnesses. Since the onset of the epidemic, approximately 40.1 million deaths have been attributed to these diseases, among a staggering 84.2 million people infected (1). These figures not only illustrate the severe impact of the epidemic but also highlight the urgent need for greater awareness. Promoting responsible sexual behavior is crucial in preventing the transmission of HIV/AIDS and other related infections.

The World Health Organization (WHO) indicates that the majority of sexually transmitted infections are asymptomatic (2). In sub-Saharan Africa (SSA), the HIV/AIDS epidemic exerts a significant toll on public health (3). In this region, the virus predominantly transmits through sexual relations, yet a considerable number of individuals do not have access to adequate prevention and treatment resources (4). A substantial proportion of new HIV infections in SSA results from unprotected heterosexual intercourse, particularly among those with multiple sexual partners. This pattern remains the primary driver of HIV transmission in the region (5).

Aphrodisiacs are substances designed to boost sexual desire (6). They can come from natural sources, like plants or animals, or be created synthetically. People often use these substances to increase their libido. However, it's important to note that many medically prescribed aphrodisiacs are sometimes misused for recreational purposes (7, 8). In fact, numerous healthy and sexually active men take them in hopes of improving their sexual performance (9).

Furthermore, most of these products have negative health effects on consumers beyond their intended treatment (10) and contribute to sexual dysfunctions (7). They can also lead to other serious health risks, such as kidney diseases, cardiac arrests, and strokes. In addition, herbal products, which often lack clear dosage instructions, expose users to risks of overconsumption due to trial and error (10).

In 2022, people who inject drugs accounted for 8% of new HIV infections (11). The relative risk of acquiring HIV was estimated to be 14 times higher among people who inject drugs than among the remainder of the adult population aged 15–49 years (12), and more than three-quarters of people who used drugs were men (11). In 2023, the median HIV prevalence among people who inject drugs in 47 reporting countries was estimated at 5% (range: 0–32%) (11), substantially exceeding the estimated 0.7% HIV prevalence in the global adult population aged 15–49 years (13); among men who inject drugs, this prevalence may reach 9% (11).

Many studies from different places have shown a clear link between using certain substances, like illegal drugs, and engaging in risky sexual behaviors. The literature also points to a higher risk of contracting sexually transmitted infections, including HIV (14–17). For instance, using sildenafil or consuming drugs and alcohol while being sexually active is connected to having more sexual partners and taking more risks during sex (18). Additionally, the use of aphrodisiacs has been associated with unprotected anal sex with partners whose HIV status is unknown, which is a significant risk for spreading HIV (15, 19).

The relationship between the intake of aphrodisiacs and engagement in high-risk sexual behaviors has been increasingly documented in existing research. Nonetheless, a notable limitation highlighted by many scholars is the small sample sizes or the restricted geographical regions examined (17), along with the highly specific demographics of the targeted populations. Consequently, the degree to which these findings can be extrapolated to more heterogeneous groups remains ambiguous. In most studies, the relation between aphrodisiac consumption and risky sexual practices is predominantly observed within homosexual and bisexual cohorts (20–22). Given that this research has focused on diverse populations, there is still a lack of comprehensive understanding regarding the relation between aphrodisiac use and risky sexual behaviors among both men in relationships and those who are single.

The sexual risk behaviors analyzed in this study pertain to activities that heighten men's vulnerability to sexually transmitted infections (STIs) and HIV. According to the latest Demographic and Health Survey (DHS) conducted in the Democratic Republic of the Congo (DRC) (23), 38% of men aged 15–59 reported having engaged in sexual activity with individuals who were not their spouses or regular partners. Notably, only 20% of these men employed condoms during such encounters (23). This finding underscores the substantial risk that the Congolese population faces regarding the transmission of HIV and other STIs. Furthermore, DRC is identified as one of the Sub-Saharan African countries with a non-negligible prevalence of HIV, currently estimated at 1% (23). The number of people living with HIV is projected to increase from 578.1 thousand in 2019 to 612.8 thousand in 2024. In addition, people who inject drugs accounted for 3.9% of all HIV infections in 2019 (13).

This analysis is based on data from a survey conducted in Kinshasa, Democratic Republic of Congo, between July and August 2020. The survey collected information from a heterogeneous sample of sexually active males aged 15–49 years on the use of aphrodisiacs and sexual practices. The study focused on adolescents and adults and pursued the following objectives: (a) to assess the risk of contracting STIs among men aged 15–49 years, irrespective of marital status; (b) to determine the association between aphrodisiac use among men aged 15–49 years and engagement in risky sexual behaviors that increase the likelihood of contracting STIs, and to compare these association between partnered and single men; and (c) to examine the interaction between aphrodisiac use and marital status, as well as between aphrodisiac use and age.

To our knowledge, this study represents the first analysis conducted in Kinshasa, a city where the proliferation of indigenous products is becoming concerning. This situation is evidenced by a multiplication of sales points offering traditional aphrodisiacs as well as products from the modern pharmaceutical industry, often sold without a medical prescription, and by a proliferation of healers and advisors operating without oversight from relevant authorities. Furthermore, more than half (56.5%) of men aged 15 to 49 reported having consumed aphrodisiacs at some point in their lives, while the prevalence over the past 12 months was estimated at 44.9% in 2020 (8). Although the overall HIV prevalence in this setting was estimated at 0.8% in 2024, according to UNAIDS statistics (13), this aggregate indicator conceals substantial spatial heterogeneity across municipalities, with prevalence estimates reaching 3.1% in Nsele and 5.4% in Gombe. However, it remains difficult to obtain, for this country, reliable and high-quality serological data regarding the serological status of its population (24).

This study presents two primary hypotheses related to the specific objectives above: (1) individuals engaging in sexual activities while under the influence of aphrodisiacs exhibit an increased susceptibility to engaging in risky sexual behaviors, which may result in STIs; (2) individuals consuming aphrodisiacs who are not in committed relationships are more likely to be exposed to these risky sexual behaviors than their counterparts who are single.

## Method and materials

### Data source

The data used in this study are secondary data obtained from a 2020 survey database on aphrodisiac consumption in Kinshasa, funded by the Bureau d'Étude et de Gestion de l'Information Statistique (BEGIS) (8). This research was conducted as a cross-sectional survey with a single wave of data collection. The database comprises information on 713 participants aged 15–49 years (irrespective of marital status), residing in Kinshasa, concerning aphrodisiac use among men and various sexual behaviors, including condom use, encounters with casual partners, frequency of sexual activity, number of sexual partners, and the nature of relationships with these partners. The study procedures and comprehensive details on data collection have been reported previously (8).

Only data from participants who were sexually active at the time of the survey, or who had been sexually active within the preceding 12 months, were included in the analysis. Of the 713 initial observations, 59 participants who had not been sexually active in the last 12 months were excluded, resulting in a final analytic sample of 654 participants. These 654 participants had complete information on all variables of interest, as detailed in the following section.

### Study variables

#### Dependent variable

In this study, the dependent variable analyzed through regression is identified as “risky sexual behavior.” This variable is a composite binary measure, assigned a value of 1 for “Yes” and 0 for “No”. The operationalization of the risky sexual behavior variable was based on an algorithm incorporating four distinct constituent indicators: (a) condom use during the most recent sexual intercourse (yes, no), (b) engagement in sexual intercourse with casual partners (yes, no), (c) the type of relationship with the partner during the most recent sexual intercourse, and (d) current marital status. The rationale for selecting these specific indicators to define risky sexual behavior is consistent with the indicators recommended by UNAIDS and those employed in previous studies (25–31).

The variable “risky sexual behavior” was operationalized differentially according to participants' marital status. Among single men, individuals were classified as engaging in risky sexual behavior if they reported either (i) sexual intercourse with a casual partner during the reference period or (ii) unprotected sexual intercourse at their most recent sexual encounter with non-cohabiting partner. Among partnered men, risky sexual behavior was defined as either (i) sexual intercourse with a casual partner or (ii) unprotected sexual intercourse during the most recent sexual encounter with someone other than their regular partner. Participants who met at least one of these criteria were categorized as engaging in risky sexual behavior (coded as 1), whereas all remaining participants were categorized as not engaging in risky sexual behavior (coded as 0).

#### Independent variable

The principal independent variable in this study is “aphrodisiac consumption in the 12 months preceding the survey.” This variable is operationalized as a dichotomous measure, coded as 1 for “Yes” (i.e., respondents who reported consuming aphrodisiacs within the last 12 months) and 0 for “No” (i.e., respondents who reported no aphrodisiac use during that period).

#### Control variables

The sociodemographic variables included as covariates in the study were marital status, educational attainment, religious affiliation, employment status, and age. Marital status was dichotomized into two categories, following (30, 32): (i) in union and (ii) single. The original variable comprised four categories: never married, partnered/married, separated/divorced, and widowed. Educational attainment (i.e., highest diploma obtained, if any) was categorized into two groups, consistent with (32): (i) lower education and (ii) higher education. The original educational variable consisted of four categories: (i) no education, (ii) primary, (iii) secondary, and (iv) higher education. The first two categories (no education and primary) were merged into “lower education,” whereas secondary and higher education were combined into “higher education.” Religious affiliation (33) comprised three categories: (i) Christian, (ii) other religions, and (iii) no religion, consistent with previous research in SSA (34). The final variable was coded as follows: 1 = Christian; 2 = “Other religions” (Kimbanguist, Muslim, Jehovah's Witnesses, Branhamist, or any other religion); 3 = no religious affiliation. Employment status (35) was classified into five categories, following the coding used in the original database (8): (i) unemployed or student, (ii) merchant/informal worker, (iii) artisan/laborer, (iv) public/private sector employee, and (v) other professions. Age was included as an additional confounding variable (36, 37). Although the original measure variable was continuous, the variable Age was dichotomized into 15–34 and 35–49 years to differentiate younger from older men within the reproductive age range. This categorization was informed by previous research (38) demonstrating that sexual behaviors and the risk of HIV and other STIs vary substantially across these age strata. For instance, the DHS analytical report indicates that HIV prevalence among men typically peaks at ages 35–39, including in the DRC (1.8%) (38), suggesting that men aged ≥35 years represent a distinct epidemiological subgroup.

The control variables implemented in this study aimed to mitigate potential confounding biases. These covariates were selected based on their empirical documentation in prior research (19, 30, 32, 33, 36, 37). Among these, factors such as age and marital status were also examined for their interactions with the independent variable.

To evaluate the risk to exposure to risky sexual behavior related to sexually transmitted infections, the following variables were considered: the use of condoms during the most recent sexual intercourse, the nature of sexual partners involved in that intercourse, the type of sexual partners engaged with when an aphrodisiac was used, and the incidence of sexual encounters with casual partners within the 12 months leading up to the survey.

### Data analysis

The sociodemographic attributes of the participants were analyzed using both absolute counts (*n*) and relative frequencies expressed as percentages (%). The primary findings concerning exposure to the risky sexual behavior related to STIs and HIV/AIDS, along with the sexual behaviors exhibited by the participants, were assessed using percentage values (%) and 95% confidence intervals (CI).

To explore the statistical relationships among aphrodisiac use, sociodemographic variables, and engaging in risky sexual behaviors, bivariate analysis was performed to determine the crude effects. A *p*-value of less than 5% (*p* < 0.05) was deemed indicative of statistical significance.

Subsequently, multivariate analysis models were employed to assess the net impact of the primary explanatory variable on the dependent variable. Linear probability models (LPM) were utilized, with risky sexual behavior designated as the outcome variable and consumption of aphrodisiacs as the primary explanatory variable. The LPM was selected due to its capacity to overcome several challenges associated with the logistic regression model, particularly the issue of unobserved heterogeneity when comparing multiple models (39), as in our case where we compare groups such as partnered men and single men. We acknowledge, however, that a well-documented limitation of the LPM is that the predicted probabilities may exceed 1 or fall below 0—values that are theoretically impossible—as well as the presence of heteroscedasticity. Nonetheless, Mood (39) contends that this limitation is generally not a major concern, especially when weighed against the disadvantages of alternative modeling approaches discussed above. Prior to estimating the model, we conducted a heteroskedasticity diagnostic using the Breusch–Pagan/Cook–Weisberg test (40). In the presence of heteroskedasticity, we addressed this issue by specifying the “robust” option at the end of the linear regression command in Stata, thereby obtaining heteroskedasticity-consistent (Eicker–White) standard errors (41). These models were adjusted for potential confounding factors (42), including age, educational attainment, marital status, employment situation, and religious affiliation.

While there is an implicit assumption that the primary variable of interest operates independently of other variables, this assumption is not invariably valid. Consequently, it was essential to account for potential interaction effects with other variables (42, 43). Interaction terms between aphrodisiac use and selected covariates (age group and marital status) were therefore introduced into the linear probability model and evaluated using Wald tests. Specifically, an interaction term was assessed to reflect the combined influence (43) of aphrodisiac consumption and marital status on the outcome variable, followed by an evaluation of the joint effect of aphrodisiac consumption and age. Interaction terms that reached statistical significance (p < 0.05) were retained in the final model specification.

Ultimately, the analyses were stratified into two distinct groups to facilitate a comparison of results between individuals in unions and those who are not. Fixed effects, represented as adjusted coefficients with 95% confidence intervals, were utilized to estimate the relationship between the independent and dependent variables. Statistical significance was determined using a *p*-value threshold of < 5% (*p* < 0.05). All statistical analyses were performed using Stata 18 software. The maps are produced using RStudio software.

## Results

The sociodemographic characteristics of the participants are summarized in Table 1. The average age of the respondents was 28 years, and at least three-quarters of the participants were not in a union at the time of the survey.

**Table 1:** Prevalence of aphrodisiac consumption and sociodemographic characteristics of participants.

Sociodemographic characteristics of participants	*N* = 654^a^	Aphrodisiac using^b^
Marital status		
Partnered	160 (24.46)	93 (58.13)
Single	494 (75.54)	226 (45.75)
Religion		
Christian	510 (77.99)	243 (47.65)
Other religion	108 (16.51)	51 (47.22)
No religion	36 (5.50)	25 (69.44)
Main occupation		
No occupation/student	193 (29.51)	83 (43.01)
Trader/hustler	196 (29.97)	98 (50.00)
Artisan/laborer	88 (13.46)	44 (50.00)
Public/private sector worker	79 (12.08)	41 (51.90)
Other occupations	98 (14.98)	53 (54.08)
Age of respondent		
Young (15–34)	513 (78.44)	236 (46.00)
Older (35–49)	141 (21.56)	83 (58.87)
Education		
Lower	84 (12.84)	36 (42.86)
Upper	570 (87.16)	283 (49.65)
District of residence		
Funa	165 (25.23)	58 (35.15)
Lukunga	155 (23.70)	84 (54.19)
Mont-Amba	155 (23.70)	86 (55.48)
Tshangu	179 (27.37)	91 (50.84)
Total	654 (100.0)	319 (48.78)

^a^Statistics presented in the columb: *n*(%).

^b^Statistics presented in the row: *n*(%).

Regarding the prevalence of aphrodisiac use (Table 1), nearly half (48.78%) of sexually active men reported consumption within the preceding 12 months. Elevated prevalence was observed among men in unions (58.13%), those reporting no religious affiliation (69.44%), older men (58.87%), and individuals with higher levels of formal education (49.65%).

**Figure 1:**
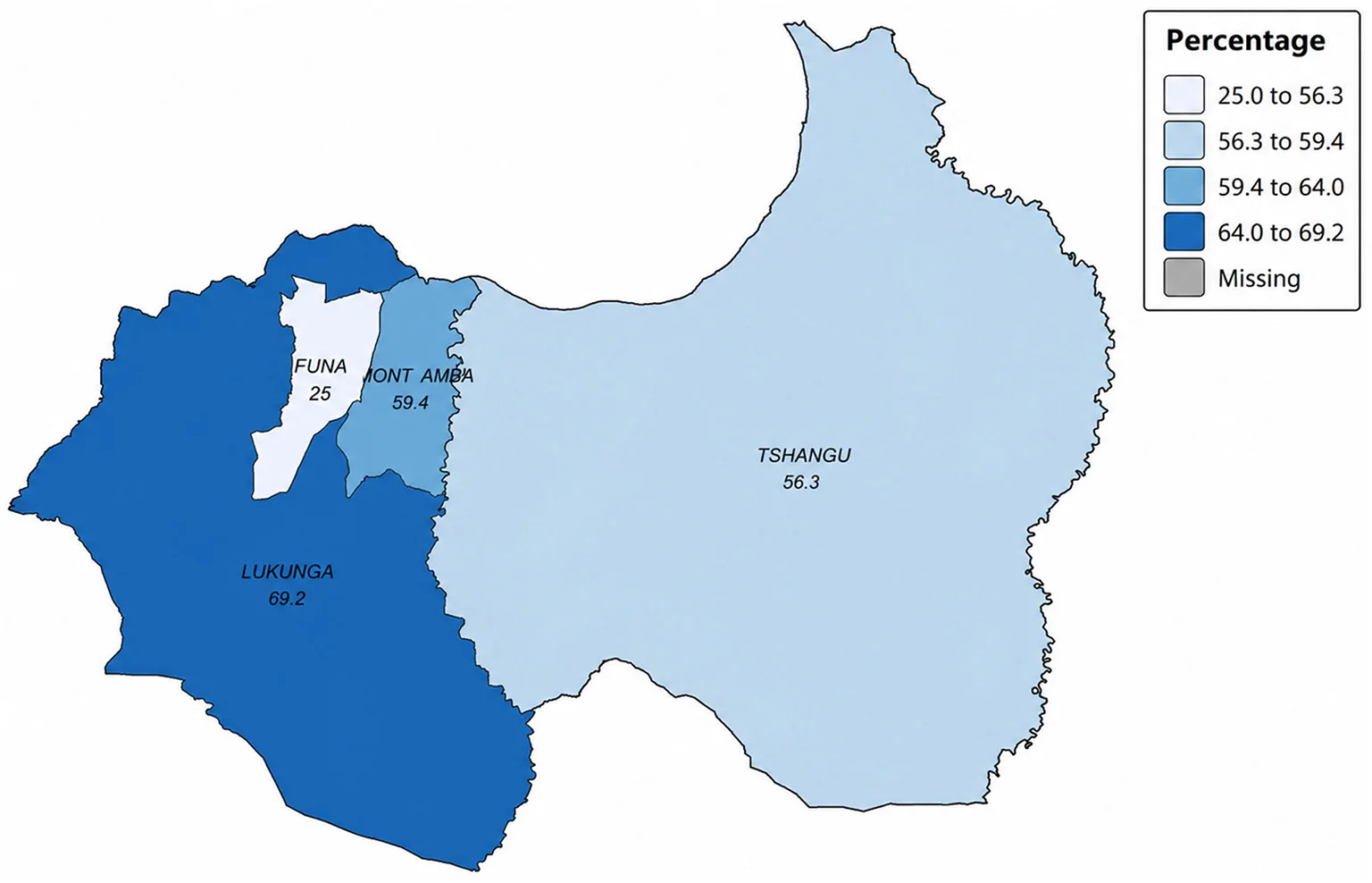
Spatial distribution of aphrodisiac consumption among partnered men.

**Figure 2:**
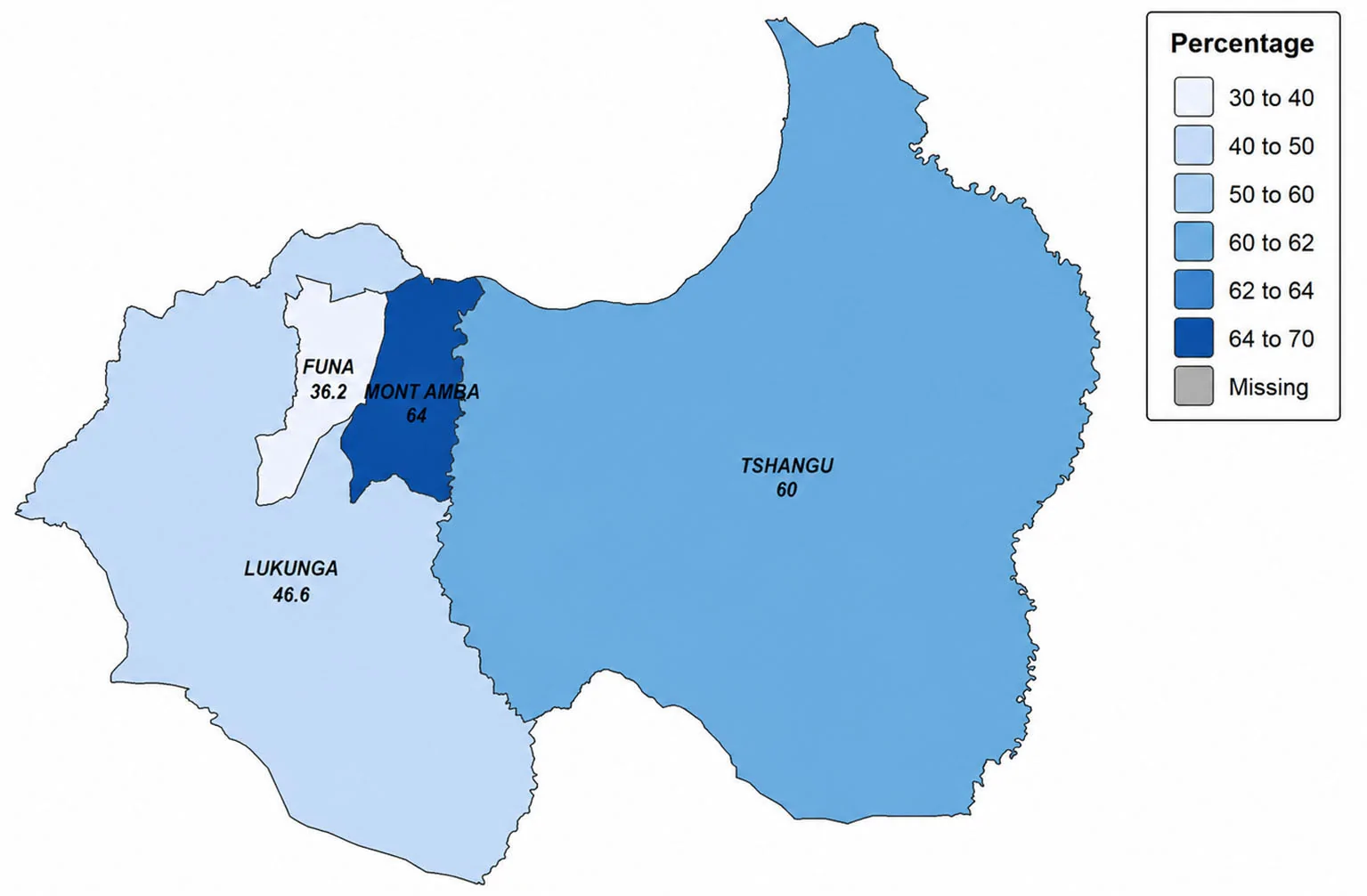
Spatial distribution of aphrodisiac consumption among single individuals.

The spatial distribution of aphrodisiac use demonstrated pronounced heterogeneity: among partnered men, the highest prevalence was observed in the district of Lukunga (69.2%), whereas the lowest was recorded in Funa (25%) (Figure 1). Among single men (Figure 2), higher levels of consumption were documented in the districts of Mont-Amba (64%) and Tshangu (60%).

### Exposure to the risk of sexually transmitted infections

According to Table 2, the results indicate that, in general, one in five men reported having had sexual intercourse with casual partners or sex workers. As might be expected, single men (21.66%) are more likely to have sex with casual partners than men in union (15.63%) Another interesting result is that 77.55% of single men reported having sex with girlfriends who do not live with them, and 4 out of 494 stated that the partner was a wife.. Meanwhile, 80.63% of men in unions reported having sex with their wife or partner living with them, while 6 out of 160 (3.75%) reported having sex with a girlfriend who does not live with them.

**Table 2:** Distribution of respondents by type of sexual partners during last sexual intercourse, disaggregated by marital status.

	Frequency	Wife or partner living with the respondent	Girlfriend not living with the respondent	Casual encounter/sex worker	
Type of sexual partners during last sexual intercourse					
Marital status	*N*	*n* (%)	*n* (%)	*n* (%)	*P*-value
Partnered	160	129 (80.63)	6 (3.75)	25 (15.63)	< 0.001
Single	494	4 (0.81)	383 (77.53)	107 (21.66)	
Total	654	133 (20.34)	389 (59.48)	132 (20.18)	
Type of sexual partners during last sexual intercourse with an aphrodisiac					
Marital status	*N*	*n* (%)	*n* (%)	*n* (%)	*P*-value
Partnered	116	58 (50.00)	8 (6.90)	50 (43.10)	< 0.001
Single	284	1 (0.35)	152 (53.52)	131 (46.13)	
Total	400	59 (14.75)	160 (40.00)	181 (45.25)	

Regarding the type of relationship with the partner during the last sexual intercourse where an aphrodisiac was consumed, the results show that 45.25% of cases had sex with casual partners. Disaggregated data by marital status reveal that: 43.10% of men in unions reported using aphrodisiacs with a casual partner, compared to 46.13% among men not in unions. 50% of men in unions stated that they had used these substances with their cohabiting spouse, compared to 6.9% with their girlfriends. In contrast, among men not in unions, a little over half (53.52%) reported having used aphrodisiacs during sexual intercourse with their girlfriends.

Table 3 shows that half (51% [95% CI: 47.15–54.84]) of respondents reported having had sexual intercourse with casual partners in the 12 months preceding the survey. This practice appears to be more common among respondents not in a union, where a little over half (52.74% [95% CI: 48.32–57.16]) of men in this subgroup engaged in sexual intercourse with one or more casual partners over the past 12 months, compared to 45.63% [95% CI: 37.82–53.43] among those in a union, but this difference is not statistically significant. Additionally, 30.47% [95% CI: 26.93–34.01] of respondents reported using a condom. This proportion was slightly higher among respondents not in a union, at 36.15% [95% CI: 30.28–42.03], compared to 17.81% [95% CI: 8.82–26.80] among their counterparts in a union.

**Table 3:** Percentage of respondents by selected variables according to their sexual behavior, disaggregated by marital status.

	*Frequency*	Used a condom during last sexual intercourse		Engaged in sexual intercourse with at least one casual partner in the past 12 months	
Marital status	*N*	%	CI95%	%	CI95%
Partnered	160	17.81	(8.82–26.80)	45.63	(37.82–53.43)
Single	494	36.15	(30.28–42.03)	52.74	(48.32–57.16)
Total	654	30.47	(26.93–34.01)	51.00	(47.15–54.84)

### Multivariate analysis

The findings from the linear probability models are summarized in Table 4, indicating that the consumption of aphrodisiacs is linked to engaging in risky sexual behaviors associated with sexually transmitted infections among men aged 15 to 49. This relationship was established in the unadjusted model (0.19 [95% CI: 0.12–0.25; *p*-value < 0.001]); however, a modest increase of approximately 3% in the risk of exposure was noted after controlling for variables such as age, marital status, education level, employment status, and religion. Specifically, men who reported consuming aphrodisiacs within the previous year exhibited a heightened risk (aCoef = 0.22 [95% CI: 0.17–0.28; *p*-value < 0.001]) of partaking in risky sexual behaviors linked to sexually transmitted infections, in contrast to those who did not engage in such consumption.

**Table 4:** Linear Probability Models of the association between aphrodisiacs consumption and sexual behavior linked to the risk of sexually transmitted infections.

Models	Unadjusted coefficients (95% CI)	*p*-value	Adjusted coefficients (95% CI)^a^	*p*-value
Aphrodisiac consumption				
Model 1: entire sample	0.19 (0.12; 0.25)	< 0.001	0.22 (0.17; 0.28)	< 0.001
Model 2: single	0.14 (0.08; 0.20)	< 0.001	0.14 (0.09; 0.20)	< 0.001
Model 3: partnered	0.47 (0.33; 0.61)	< 0.001	0.48 (0.33; 0.62)	< 0.001

^a^Adjusted for: marital status, age, professional status, education, religion and district of residence.

**Figure 3:**
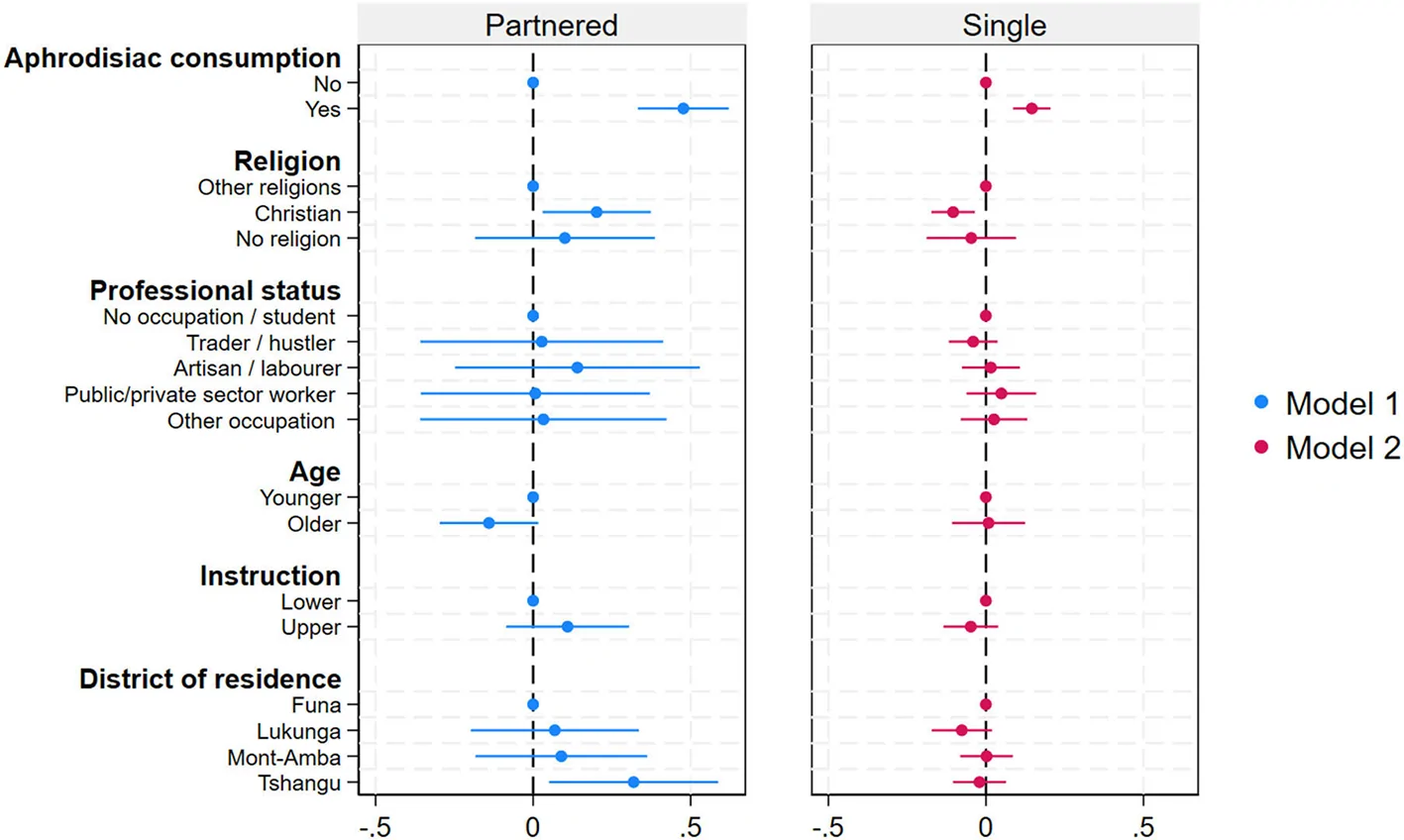
Linear probability Models with risky sexual behavior as outcome variable (stratified by marital status).

Moreover, the findings from the stratified analyses based on marital status (see Table 4 and Figure 3) reveal that men in unions who engage in the consumption of aphrodisiacs exhibit a significantly greater risk of contracting STIs compared to their non-union counterparts who also consume aphrodisiacs. Furthermore, the outcomes from Model 2, as outlined in Table 4, demonstrate a heightened risk (aCoef = 0.14 [95% CI: 0.09–0.20; *p*-value < 0.001]) of engaging in risky sexual behaviors associated with STIs among aphrodisiac consumers relative to non-consumers within the group of men not in unions. Notably, this risk of exposure to STIs escalates threefold for those who are married or in consensual unions while consuming aphrodisiacs (Model 3), reaching a coefficient of 0.48 [95% CI: 0.33–0.62; *p*-value < 0.001].

**Figure 4:**
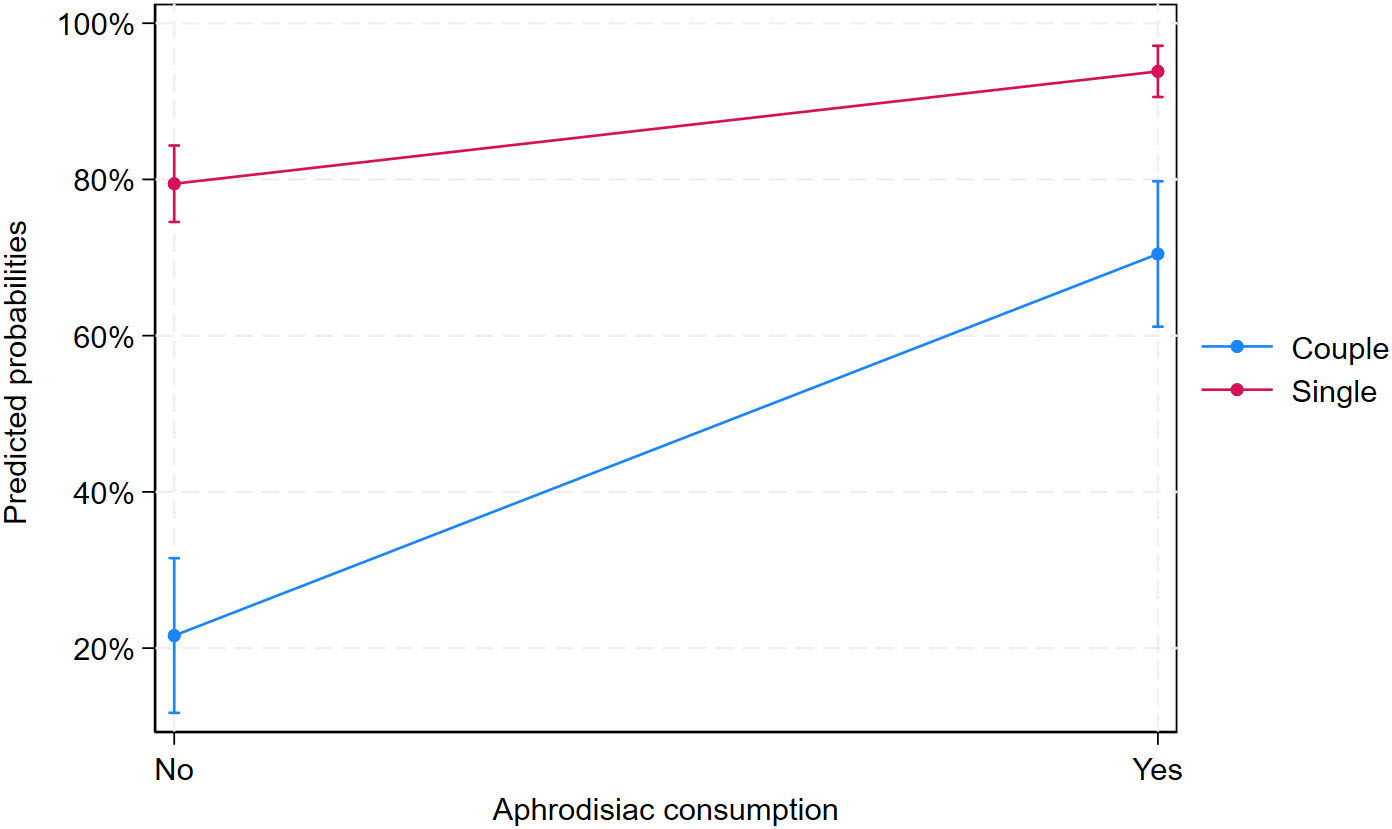
Predicted probabilities of risky sexual behavior related to sexually transmitted infections: interaction term between aphrodisiac consumption and marital status.

Table 5 presents the results of two models with interaction terms. The interaction between aphrodisiac consumption and marital status is significant (aCoef = 0.34, 95% CI = 0.19–0.49). The predicted probabilities of engaging in risky sexual behavior related to STIs, based on aphrodisiac consumption and marital status, are illustrated in Figure 4. This figure shows that men not in unions who consume aphrodisiacs have a slightly higher risk (90%) of engaging in risky sexual behavior related to STIs, compared to 80% among non-consumers. However, among men in unions, the probability of engaging in risky sexual behavior related to STIs is even higher, increasing from approximately 20% for non-consumers to 70% for consumers.

**Table 5:** Linear probability Models of the association between aphrodisiac consumption and sexual behavior linked to the risk of sexually transmitted infections. With interaction term.

Models	Variable	Coef. ^(a)^	95% CI
Model 4	Aphrodisiac consumption	0.14^***^	(0.8; 0.20)
	Marital status (couple)^(**b)**^	−0.55^***^	(−0.69; −0.42)
	Aphrodisiac consumption ^*^Marital status (Partnered)	0.34^***^	(0.19; 0.49)
Model 5	Aphrodisiac consumption	0.16^***^	(0.10; 0.22)
	Age (older 35–49)^(**c)**^	−0.21^**^	(−0.35;−0.72)
	Aphrodisiac consumption ^*^Age (Older 35–49)	0.28^***^	(0.13; 0.43)

^**(a)**^Adjusted for: marital status, age, professional status, education, religion and district of residence.

^**(b)**^The reference category is single.

^**(c)**^The reference category is younger (15–34).

^***^p < 0.001; ^**^p < 0.05.

Similarly, for the age variable, a significant interaction was observed between aphrodisiac consumption and age (aCoef = 0.28, 95% CI = 0.13–0.43). The predicted values for the probability of engaging in risky sexual behavior related to STIs, presented in Figure S1, indicate a higher probability of risky behavior among older individuals (35–49 years) who consume aphrodisiacs, compared to non-consumers, increasing from 30% (non-consumers) to 75% (consumers). In contrast, the increase in the probability of engaging in risky sexual behavior related to STIs is much smaller among younger individuals (15–34 years), with 90% for consumers and 75% for non-consumers.

## Discussions

To the best of our understanding, this study constitutes the first exploration of the association between aphrodisiac consumption and engagement in risky sexual behaviors associated to STIs, including HIV infections, in Kinshasa, DRC. The research examined a diverse sample of 654 males, aged between 15 and 49 years, irrespective of their marital status or sexual orientation.

While previous studies conducted in various contexts have implicitly indicated the presence of an independent correlation between aphrodisiac use and risky sexual behaviors (15, 44, 45), this research specifically has provided empirical evidence of a meaningful interaction between aphrodisiac consumption and both marital status and age. Additionally, this study enriches our knowledge on the link between aphrodisiac use and risky sexual behaviors associated with sexually transmitted diseases and infections by utilizing a more diverse population and without making distinctions based on sexual orientation.

### Exposure to the risk of sexually transmitted infections

The study has revealed a notably high level of STIs risk exposure among males aged 15–49, with men not in unions exhibiting greater risk compared to those in relationships.

More than half of the respondents indicated they had engaged in sexual intercourse with casual partners or sex workers within the 12 months prior to the survey, and one in five men reported such encounters during their most recent sexual activity. This pattern aligns with findings from the 2024 DHS conducted in DRC (23), which estimated that 46% of men in Kinshasa had engaged in sexual intercourse in the past 12 months with individuals who were neither their spouses nor cohabiting partners.

Moreover, the study noted a low prevalence of condom use during the last sexual encounter, estimated at approximately 30% among participants. This low prevalence was observed among both partnered men (i.e., those in unions) and non-partnered men; however, the frequency of condom use among men not in unions was approximately twice that of their counterparts in unions. This finding suggests that men in unions generally do not adopt protective measures during sexual intercourse with marital or other intimate partners.

### Aphrodisiac consumption and risky sexual behaviors

The second aim of this research was to investigate the relationship between the consumption of aphrodisiacs among men aged 15 to 49 and the prevalence of risky sexual behaviors, with a comparative analysis conducted between men who are in unions and those who are not.

Employing Linear Probability Models that account for possible confounding variables, a significant relationship was identified between the intake of aphrodisiacs and engagement in risky sexual behaviors associated with STIs, including HIV infection, among males aged 15–49. Specifically, men who reported consuming aphrodisiacs within the preceding year exhibited a higher likelihood of participating in such risky sexual practices compared to their counterparts who did not consume these substances.

Moreover, the percentage of participants who engaged in sexual intercourse with casual partners during their last sexual encounter increased twofold among those who had used aphrodisiacs. Comparable results have been observed in numerous studies investigating the relationship between consumption of aphrodisiacs and risky sexual practices (15, 21, 22, 44–46). These studies suggest that individuals who consume such substances are more prone to partake in risky behaviors, which include a heightened risk of HIV transmission (47–49). The use of aphrodisiacs may prolong sexual intercourse and increase the exchange of bodily fluids, thereby raising the risk of STIs transmission, including HIV (47). Furthermore, another possible explanation is that aphrodisiacs may lead to disinhibition, which could increase the likelihood of engaging in unprotected sex.

Consequently, the findings from these analyses support the initial hypothesis (H1), which posits that the intake of aphrodisiacs increases individuals' susceptibility to participating in high-risk sexual activities, including casual sexual encounters, unprotected sex, interactions with multiple partners, and extramarital affairs. Furthermore, the results, after differentiating the sample based on marital status, reveal that men who are in committed relationships and consume aphrodisiacs are three times more likely to engage in high-risk sexual behavior associated with STIs than their single counterparts who also engage in aphrodisiac consumption.

Nevertheless, this result stands in opposition to our original hypothesis (H2), which posited that single men who utilize aphrodisiacs would exhibit a higher risk compared to their counterparts who are in unions. This divergence may be attributed to the observation that a significant number of single men already partake in high-risk sexual practices (50), while men in unions might be more inclined to alter their previously safer sexual conduct following the consumption of aphrodisiacs.

### The moderating effect of marital status and age

The third objective of this study aimed to examine the interaction between the consumption of aphrodisiacs and factors such as marital status and age. The results suggest that both marital status and age significantly influence the relationship between aphrodisiac consumption and engagement in risky sexual behaviors associated with STIs.

Specifically, the analysis of the interaction between aphrodisiac use and marital status reveals that those in cohabiting unions who consume aphrodisiacs are at a notably higher risk of participating in unsafe sexual practices compared to their single counterparts. This phenomenon may be attributed to the tendency of partnered men, when consuming aphrodisiacs, to neglect preventive measures. Consequently, these substances may facilitate behaviors that markedly increase the probability of engaging in high-risk sexual activities associated with STIs among partnered men, whereas previous empirical data from the DRC (23) (reference) has shown that partnered men are generally less likely than single men to engage in high-risk sexual behaviors.

Moreover, concerning age, older men (ages 35–49) who consume aphrodisiacs exhibit a significantly heightened risk of engaging in risky sexual behaviors related to STIs when compared to younger men. This is particularly noteworthy, as older individuals are typically regarded as being more diligent in implementing preventive strategies against unsafe sexual practices (51). These findings imply that the use of aphrodisiacs may diminish the propensity of older men to adhere to protective measures against STIs, despite their anticipated greater awareness and understanding of infection prevention. However, these results do not align with those of Schumacher et al. (52), who found an association only among young people and not among adults. This difference may be due to their use of a high-risk population, including individuals in detention and experiencing homelessness.

This observation gains further importance considering that older men are generally expected to possess enhanced knowledge regarding preventive practices against infections relative to younger individuals. Additionally, this trend is corroborated by the results presented in the Table S1, which indicate that older men had a lower exposure to the risk of contracting STIs than their younger counterparts, both prior to and after adjusting for confounding factors.

This research employed a representative dataset from Kinshasa, encompassing all four districts of the city, and implemented a complex sampling design to investigate the relationship between the consumption of aphrodisiacs and risky sexual behaviors. Nonetheless, the cross-sectional nature of the data necessitates caution concerning the temporal precedence of the variables studied, as it is possible that risky sexual behaviors could also influence the consumption of aphrodisiacs.

The results of this research offer valuable perspectives on the relationship between the use of aphrodisiacs and engaging in risky sexual behaviors among men in Kinshasa. The evidence indicates that the intake of these substances, either prior to or during sexual activity, could elevate the likelihood of engaging in risky sexual behaviors associated with sexually transmitted infections. This finding corroborates the conclusions drawn from earlier studies conducted in various other settings.

This risk exists for all men, regardless of their relationship status, but it is notably more pronounced among partnered men. Additionally, our research makes a substantial contribution to elucidating the complex association between aphrodisiac use and the engagement in high-risk sexual behaviors among men. The findings indicate that the use of aphrodisiacs does not constitute an independent risk factor; rather, it exerts its effect through significant interactions with other variables, notably age and marital status. Therefore, it is essential to consider these interactions in order to achieve a comprehensive understanding and critical assessment of the associated risks.

Future investigations should additionally assess the effects of aphrodisiac consumption on other significant outcomes, such as unintended pregnancies and abortions, to enhance our comprehension of how the consumption of aphrodisiacs impact the reproductive health of men and their intimate partners.

In conclusion, it is imperative for Congolese authorities to initiate a transformative approach by developing targeted prevention strategies for this vulnerable population, in order to mitigate the ongoing and unregulated use of these substances. This population subgroup frequently exhibits the erroneous belief that these substances enhance sexual performance; however, empirical evidence indicates that they are associated with substantial adverse health outcomes.

## Data Availability

The raw data supporting the conclusions of this article will be made available by the authors, without undue reservation.
