# Correction: Do partnered male aphrodisiac users engage in higher sexual risk behaviors related to sexually transmitted infections than single men? Evidence from a survey conducted in Kinshasa, Democratic Republic of the Congo

**DOI:** 10.3389/fpubh.2026.1970599

**Published:** 2026-08-20

**Authors:** Frontiers Production Office

**Affiliations:** Frontiers Media SA, Lausanne, Switzerland

**Keywords:** aphrodisiac, human immunodeficiency virus, Kinshasa, partnered men, risky sexual behavior, sexual intercourse, sexually transmitted infections

Affiliation 1 was incorrectly spelled. It should appear as “1. Centre for Demographic Research, Université catholique de Louvain, Louvain-la-Neuve, Belgium”.

Affiliation 3 was missing a comma. It should appear as “3. Ecole des Sciences de la Population et de Développement, Université de Kinshasa, Kinshasa, Democratic Republic of Congo”.

There was a mistake in Figures 1–4 as published. The wrong images were typeset. The image for Figure 1 was the one for Figure 3, the one for Figure 2 was the image for Figure 4, the one for Figure 3 was the one for Figure 2, the one for Figure 4 was the one for Figure 1. The corrected figures and their captions appear below.

**Figure 1:**
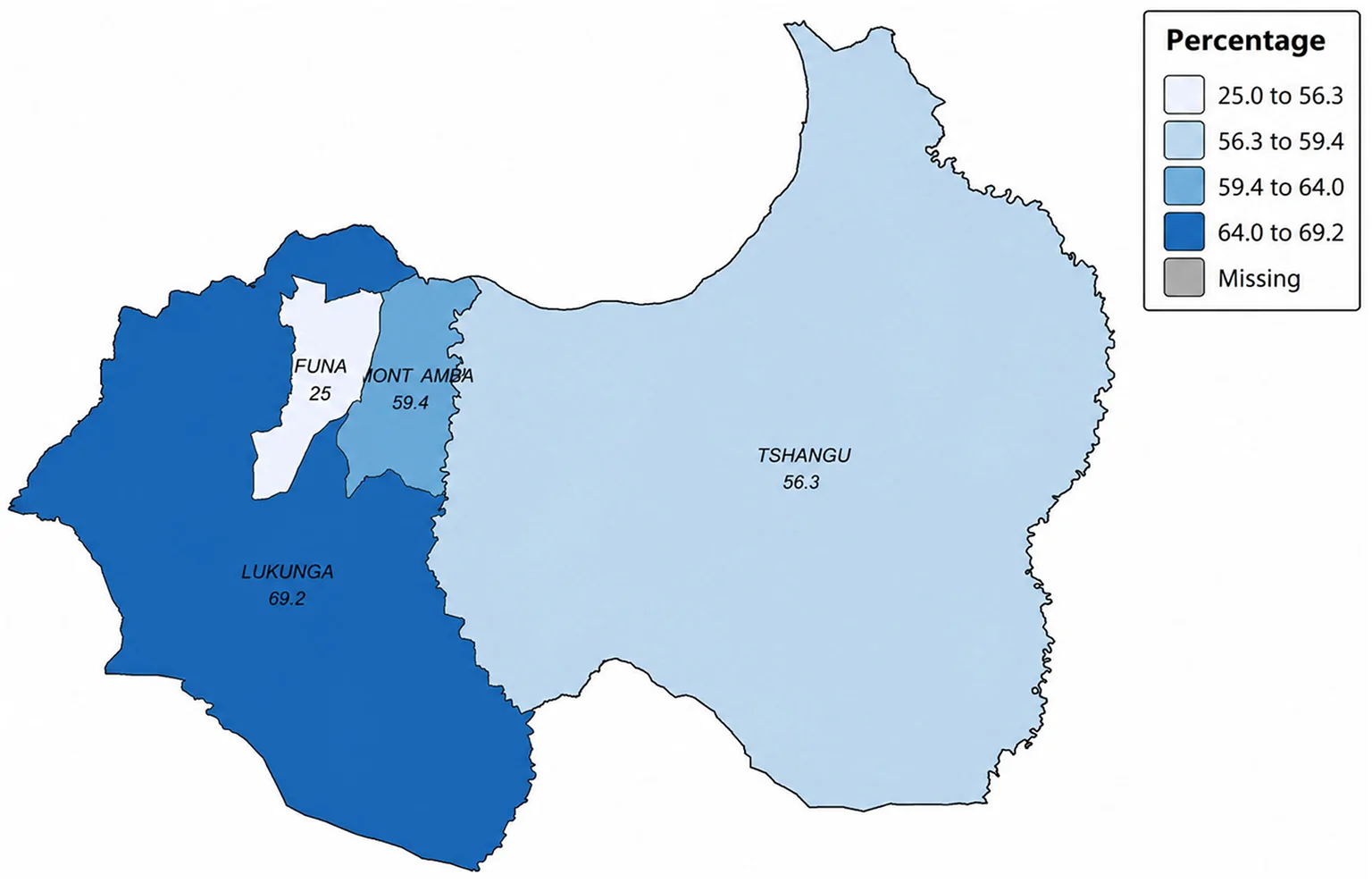
Spatial distribution of aphrodisiac consumption among partnered men.

**Figure 2:**
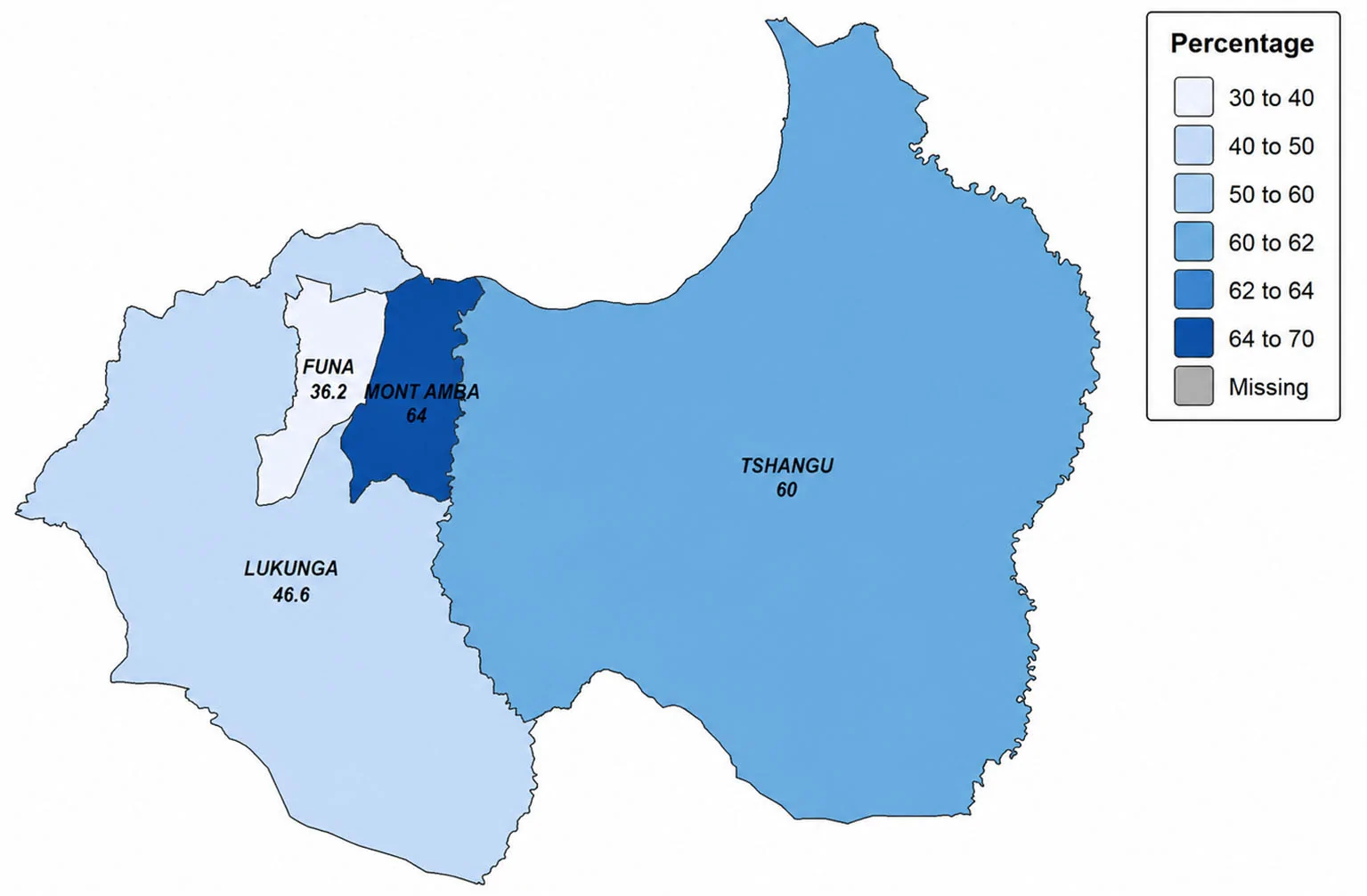
Spatial distribution of aphrodisiac consumption among single individuals.

**Figure 3:**
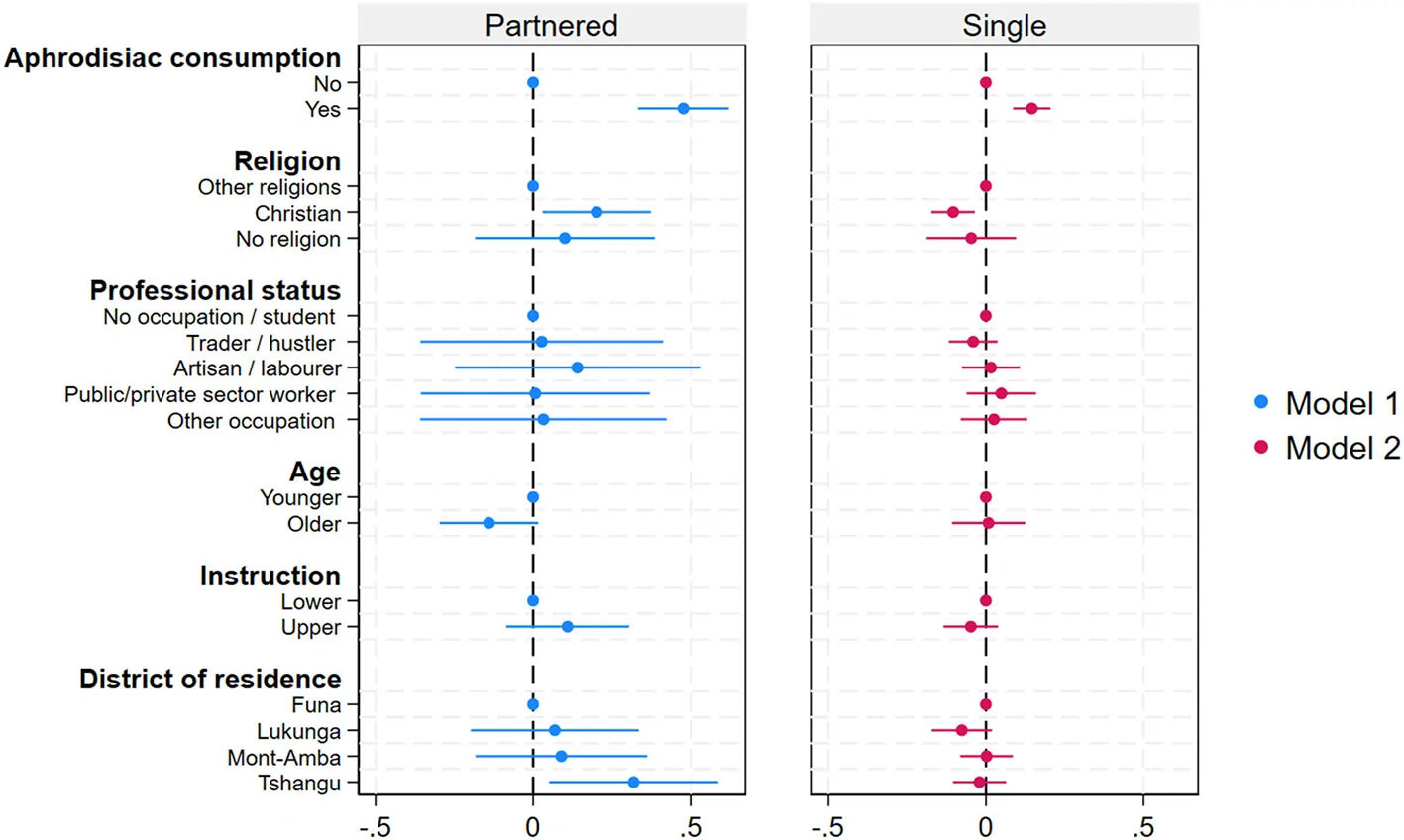
Linear probability models with risky sexual behavior as outcome variable (stratified by marital status).

**Figure 4:**
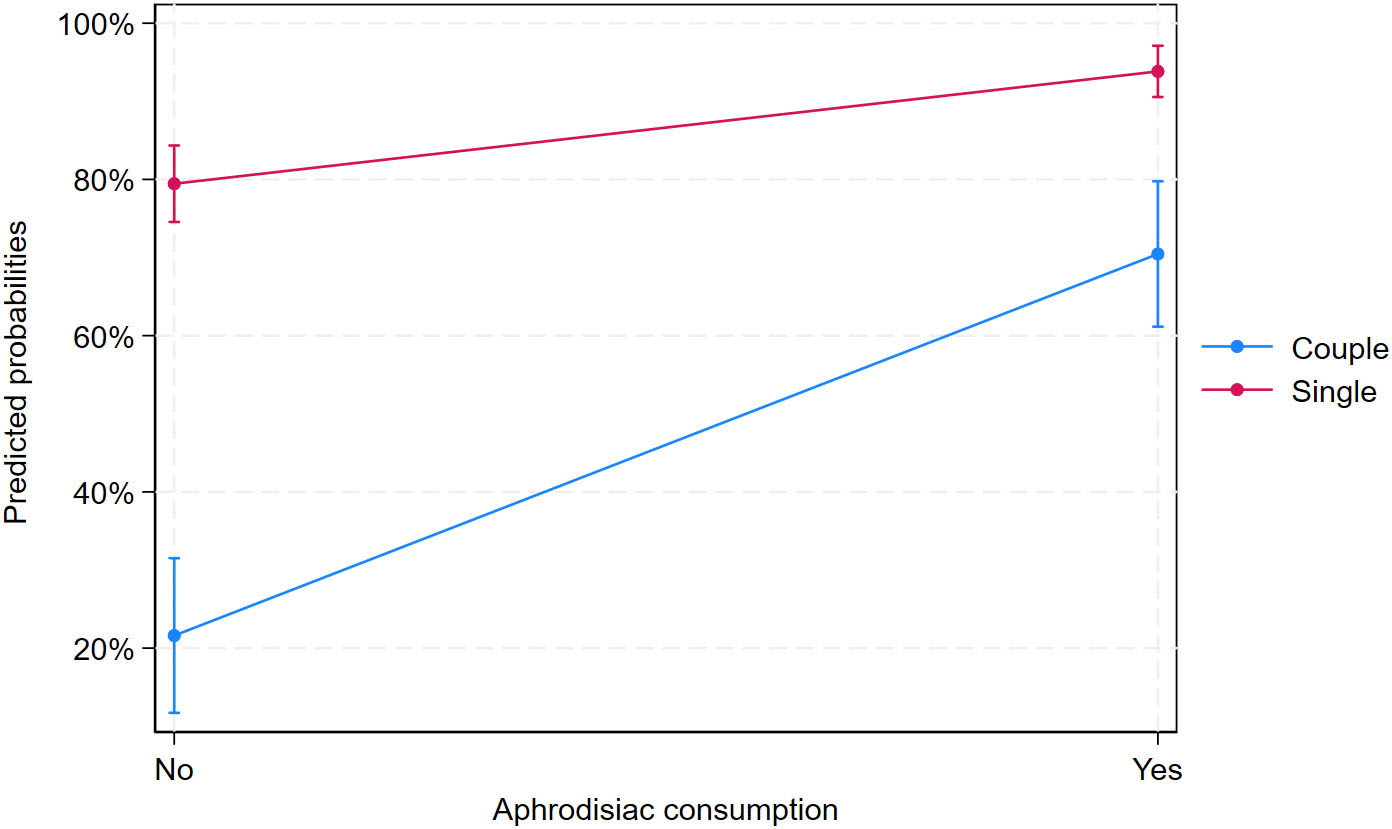
Predicted probabilities of risky sexual behavior related to sexually transmitted infections: interaction term between aphrodisiac consumption and marital status.

There is an error in Table 5. In the first row (Model 4) and last column (95% CI), a minus sign is erroneously included. The values “(−0.8; 0.20)” should be “(0.8; 0.20)”.

The original version of this article has been updated.

