# Supplementary material for: Do partnered male aphrodisiac users engage in higher sexual risk behaviors related to sexually transmitted infections than single men? Evidence from a survey conducted in Kinshasa, Democratic Republic of the Congo
Source: Front Public Health. 2026 Aug 14;14:1898629. doi: 10.3389/fpubh.2026.1898629 (PMC13540628; doi:10.3389/fpubh.2026.1898629)
Supplement: Supplementary file 1 [file Table_1.docx]

Table S1. Linear probability Models with risky sexual behavior as outcome variable (full table)

| **Variable** | **Univariate models** | |  | **Multivariate model** | |
| --- | --- | --- | --- | --- | --- |
|  | **Unadjusted coefficients**  **(95% CI)** | **p-value** |  | **Adjusted coefficients**  **(95% CI)** | **p-value** |
| **Aphrodisiac consumption** |  |  |  |  |  |
| No | Ref | - |  | Ref | - |
| Yes | 0.19 (0.12; 0.25) | 0.000 |  | 0.22 (0.17; 0.28) | 0.000 |
| **Religion** |  |  |  |  |  |
| Other religions | Ref | - |  | Ref | - |
| Christian | 0.03 (-0.05; 0.12) | 0.422 |  | -0.01 (-0.08; 0.07) | 0.851 |
| No religion | 0.06 (-0.09; 0.22) | 0.423 |  | 0.02 (-0.12; 0.17) | 0.733 |
| **Marital status** |  |  |  |  |  |
| Single | Ref | - |  | Ref | - |
| Partnered | -0.36 (-0.43; -0.29) | 0.000 |  | -0.36 (-0.46; -0.26) | 0.000 |
| **Age** |  |  |  |  |  |
| Young (15 – 34) | Ref | - |  | Ref | - |
| Older (35 – 49) | -0.26 (-0.34; -0.18) | 0.000 |  | -0.05 (-0.15; 0.05) | 0.347 |
| **Schooling** |  |  |  |  |  |
| Lower | Ref | - |  | Ref | - |
| Upper | 0.01 (-0.08; 0.11) | 0.810 |  | -0.01 (-0.10; 0.08) | 0.805 |
| **Main occupation** |  |  |  |  |  |
| No occupation/student | Ref | - |  | Ref |  |
| Trader/hustler | -0.08 (-0.16; 0.00) | 0.060 |  | -0.03 (-0.11; 0.04) | 0.392 |
| Artisan/labourer | -0.07 (-0.18; 0.03) | 0.150 |  | 0.03 (-0.07; 0.12) | 0.553 |
| Public/private sector worker | -0.23 (-0.34; -0.12) | 0.000 |  | -0.02 (-0.12; 0,08) | 0.711 |
| Other occupations | -0.10 (-0.21; -0.00) | 0.042 |  | 0.02 (-0.08; 0.12) | 0.672 |
| **District of residence** |  |  |  |  |  |
| Funa | Ref | - |  | Ref | - |
| Lukunga | -0.09 (-0.19; -0.01) | 0.038 |  | -0.05 (-0.13; 0.04) | 0.285 |
| Mont-Amba | -0.01 (-0.10; 0.08) | 0.884 |  | 0.00 (-0.08; 0.08) | 0.963 |
| Tshangu | 0.01 (-0.08; 0.10) | 0.816 |  | 0.05 (-0.03; 0.14) | 0.196 |

Ref. The reference category.
